# Ab initio structural dynamics of pure and nitrogen-containing amorphous carbon

**DOI:** 10.1038/s41598-023-46642-7

**Published:** 2023-11-11

**Authors:** Brad A. Steele, Sorin Bastea, I-Feng W. Kuo

**Affiliations:** Lawrence Livermore National Laboratory, Physical and Life Sciences Directorate, 7000 East Ave., Livermore, California 94550 USA

**Keywords:** Structure of solids and liquids, Phase transitions and critical phenomena, Atomistic models

## Abstract

Amorphous carbon (a-C) has attracted considerable interest due to its desirable properties, which are strongly dependent on its structure, density and impurities. Using ab initio molecular dynamics simulations we show that the sp^2^/sp^3^ content and underlying structural order of a-C produced via liquid quenching evolve at high temperatures and pressures on sub-nanosecond timescales. Graphite-like densities ($\lesssim$ 2.7 g/cc) favor the formation of layered arrangements characterized by sp^2^ disordered bonding resembling recently synthesized monolayer amorphous carbon (MAC), while at diamond-like densities ($\gtrsim$ 3.3 g/cc) the resulting structures are dominated by disordered tetrahedral sp^3^ hybridization typical of diamond-like amorphous carbon (DLC). At intermediate densities the system is a highly compressible mixture of coexisting sp^2^ and sp^3^ regions that continue to segregate over 10’s of picoseconds. The addition of nitrogen (20.3%) (a-CN) generates major system features similar with those of a-C, but has the unexpected effect of reinforcing the thermodynamically disfavored carbon structural motifs at low and high densities, while inhibiting phase separation in the intermediate region. At the same time, no nitrogen elimination from the carbon framework is observed above $\simeq$ 2.8 g/cc, suggesting that nitrogen impurities are likely to remain embedded in the carbon structures during fast temperature quenches at high pressures.

## Introduction

The tendency of carbon to form a variety of crystalline and disordered structures is well known, and largely attributable to its ability to have three distinct hybridizations: $sp^1$, $sp^2$, and $sp^3$. The latter two, corresponding to 3-fold and 4-fold coordination, respectively, dominate in the solid state and characterize in turn the graphite and cubic diamond allotropes. Amorphous carbon (a-C) is a common disordered material with a complex structure determined by the relative amounts of 3-fold and 4-fold coordination present in the system^1–9^, as well as density and impurities such as nitrogen (a-CN) and hydrogen (a-CH)^2,4,10–14^; the fraction of sp^3^ bonding decreases for example with density^13,15–23^. Because a-C has desirable and possibly tunable mechanical, optical, and electronic properties it has been extensively studied^13,15–44^ and structural defect engineering has emerged as a major avenue for designing a-C materials for specific applications^39,40^. a-C can be synthesized through a variety of ion beam and pulsed laser deposition techniques^13–17^; recently, monolayer amorphous carbon (MAC), a two-dimensional material, has been created using laser-assisted chemical vapor deposition^45^. a-C is also commonly produced during the detonation of negative oxygen balance high explosives^46–50^ or through the shock-compression of organic materials via mechanisms that have not been fully elucidated yet^51,52^. Molecular dynamics (MD) simulations have been carried out to model the ion beam deposition process that is often used for a-C production, usually employing a liquid quench protocol^18–20,23,30,53–57^, where liquid carbon near 5000 K is cooled to room temperature on time scales of 10’s of picoseconds. Direct deposition simulations have also been performed^56,58,59^ and lower quenching rates^36,60–62^ have been achieved using empirical potentials and machine learning potentials such as GAP^41,58,59,61,63–65^. Whereas these efforts have been primarily aimed at better understanding the structure of a-C that is typically recovered at standard temperature and pressure conditions, the kinetic processes controlling a-C formation have been less studied. A notable exception is the work of Thapa et al.^41^, which studied the formation of an amorphous graphite (a-G) material using ab initio methods up to 160 atoms and with the GAP potential up to 1000 atoms. Kinetic effects are especially important because a-C and its impurity-containing variations are metastable systems, the result of “frozen” dynamics into states whose properties are dictated by the interplay between physical and chemical processes on the one hand, and thermodynamic conditions and their rates of change on the other. It is well known for example that the phase transformation kinetics between carbon equilibrium phases has a significant pressure and temperature dependence^66–69^, while the properties of precipitating carbon-rich condensed phases have a large effect on detonation events on nanosecond to microsecond time-scales and are in turn strongly dependent on thermodynamic paths and associated transit times^48,49,70–78^. Herein we perform large-scale ab initio MD simulations of pure and nitrogen-containing (20.3%) liquid carbon (LC and LCN, respectively) quenches to an elevated temperature (3000 K) inside the solid region of the phase diagram, and analyze the evolution and properties of the resulting material over 10’s of picoseconds time scales. The results shed new light on the formation mechanisms and characteristics of a-C and a-CN, can help guide in silico material design and synthesis efforts employing efficient atomistic methodologies^79^, and may enable the development of effective models for complex phenomena such as shock-induced chemistry and detonation, which are often accompanied by the precipitation of carbon materials at high pressures and temperatures^51,52,77,78^.

## Results

**Figure 1:**
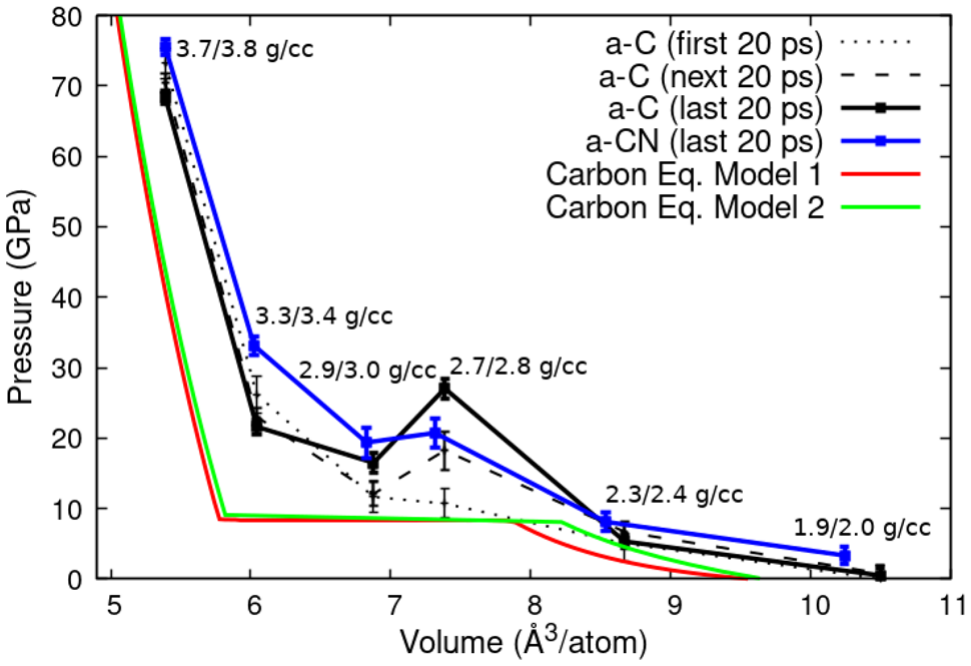
Pressure-volume relationship for amorphous carbon (a-C) quenched to 3000 K averaged over different time intervals. The error bars are the standard deviation with time for the given time interval. The curves are compared to carbon 3000 K isotherms calculated using two different thermodynamic models^73,80^.

**Figure 2:**
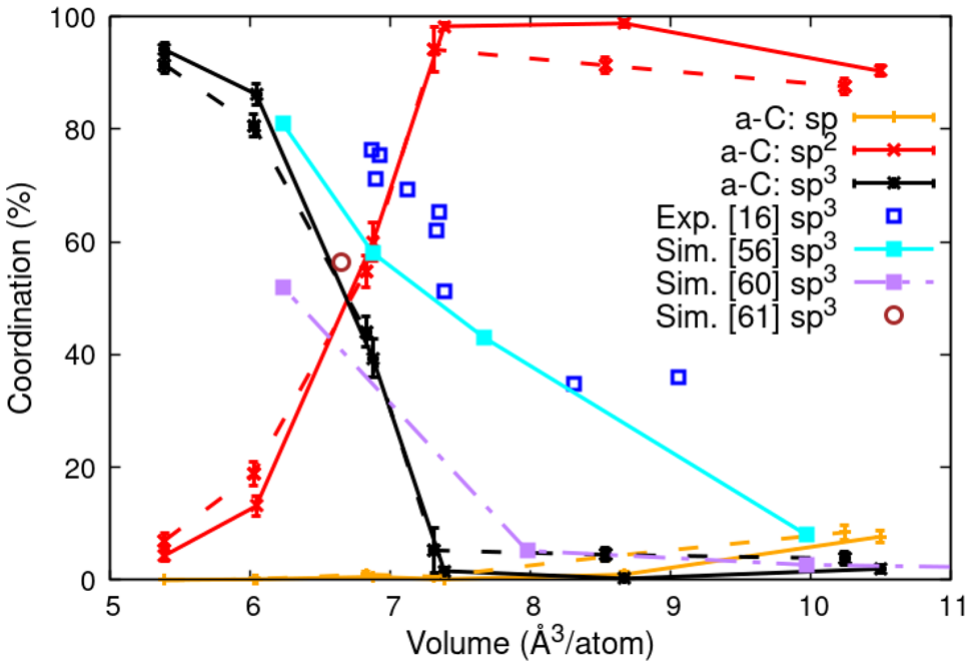
Atomic coordination of carbon atoms for a-C and a-CN (20.3 % N) vs. volume. The error bars are the standard deviation with time calculated for the final 20 ps of the simulations. The a-CN curves are displayed with dashed lines with the same color as a-C. The results are compared with ion deposition experiments (Exp.) by Fallon et al.^16^ and simulations (Sim.) by Marks et al.^56^, Ranganathan et al.^60^, and Tomas et al.^61^.

Simulations of LC (and LCN) quenched to 3000 K were performed for densities ranging between 1.9 and 3.7 g/cc (2.0 to 3.8 g/cc, respectively). Note that LC and LCN calculations correspond to the same atomic densities. The atomic coordination vs. time for all densities are provided in the Supplementary Information (SI) Figures S2 and S3. Additional simulation details are provided in the “Methods” section.

**Figure 3:**
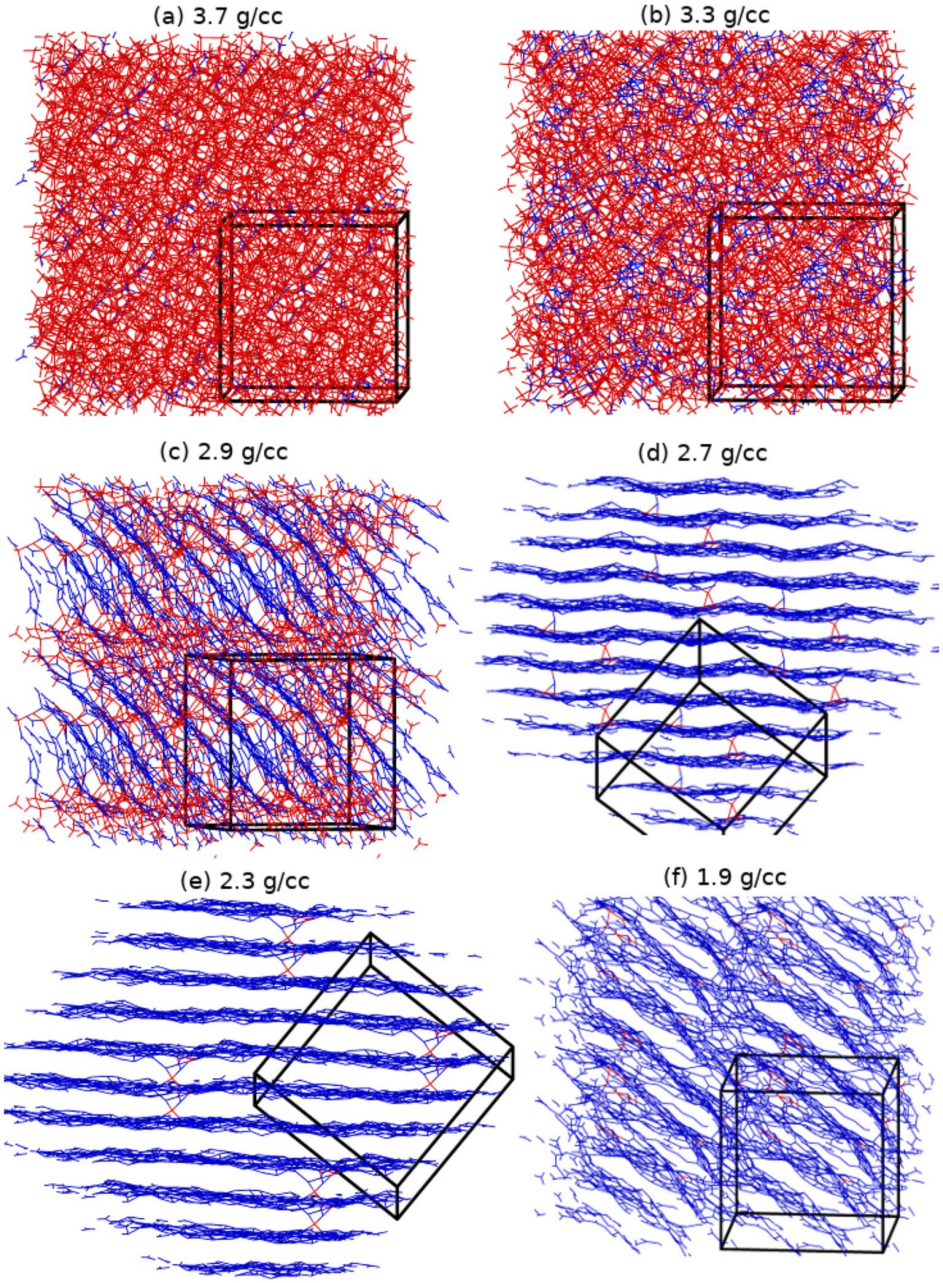
Snapshots of the final structure of a-C at 3000 K at each density studied. Atoms are color-coded based on the atomic coordination; 3-fold coordinated atoms are blue and 4-fold coordinated atoms are red. For visualization purposes, each image is a 2×2×2 supercell of the actual simulation cell (black box).

**Figure 4:**
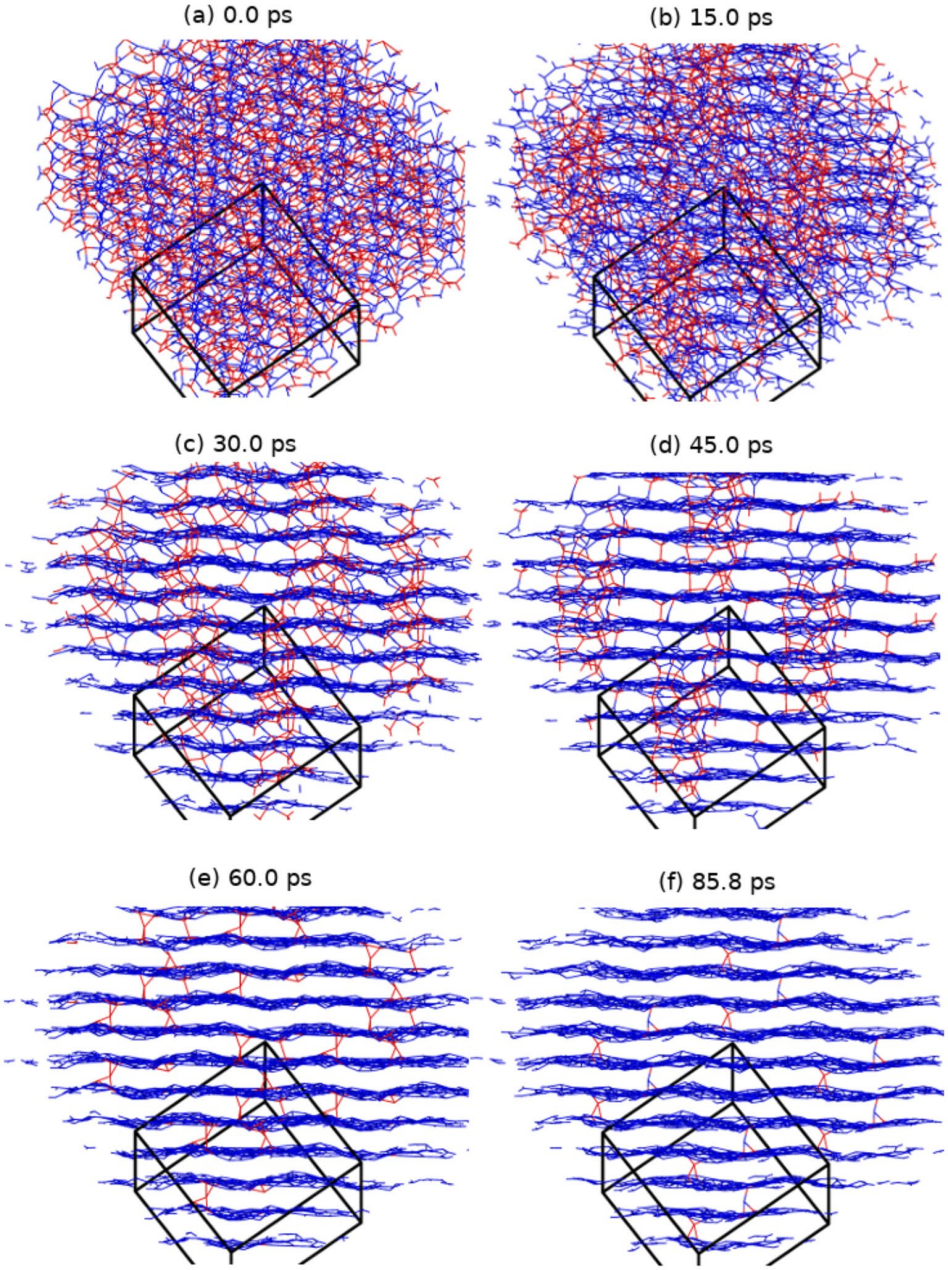
Structural evolution as a function of time of a-C at 3000 K and 2.7 g/cc at 0.0, 15.0, 30.0, 45.0, 60.0, and 85.8 ps showing the transformation to a layered structure. Atoms are color-coded based on the atomic coordination. For visualization purposes, each image is a 2×2×2 supercell of the actual simulation cell (black box).

The simulations reveal at least two distinct types of a-C structures following the temperature quench from the liquid state to the solid state region: diamond-like amorphous carbon (DLC)^2,4,10–14^ and graphite-like amorphous carbon (a-G)^41^. At high densities (and pressures) the system consists mainly of 4-fold coordinated atoms with predominantly tetrahedral local structure, while the lower densities favor 3-fold coordination in a layered motif. In the intermediate regime, the (P,V) representation of the simulation results shows van der Waals loop-like behavior associated with a first order phase transformation - see Fig. 1, which is accompanied by a sharp conversion between the two dominant coordination states, sp^3^ and sp^2^ - see Fig. 2. Indeed, experimentally validated equations of state (EOS) for carbon^73,80^ indicate that at 3000 K the diamond - graphite transformation pressure is around 10 GPa. The simulated 3000 K isotherm is shifted to higher pressures compared with the EOS prediction, and the transition pressure (which could be estimated from the P-V curve using an approximate Maxwell construction) is accordingly larger; the volume change on the other hand is smaller than the EOS one. These differences may be due to DFT approximations and system size limitations which, while important, should not affect our analysis and conclusions. The addition of nitrogen to the system has the effect of smearing the transformation between diamond-like and graphite-like behavior by noticeably reducing the volume change between these states - see Fig. 1. This is also evident, but not as pronounced, in the coordination plot as a function of density - see Fig. 2.

At high densities, where the material is primarily sp^3^ bonded, the final sp^3^ fractions observed in the simulations are consistent with room temperature DFT calculations by Marks et al.^30^ and ion beam deposition experiments^13,16^ - see Fig. 2. At low densities on the other hand they are significantly smaller. The difference with the results by Marks et al. is probably explained by the higher temperature and longer timescales of our simulations (see also below), while the disagreement with the ion beam deposition experiments may be due to the complex heating and temperature quenching profiles likely generated by ion bombardment processes, which are not modeled in these simulations^59^. Simulations by Ranganathan et al. on timescales of 100’s ps employing an empirical potential^60^ agree with the current results at low densities - see Fig. 2, but underpredict the sp^3^ fraction at high densities, possibly due to limitations of the empirical potential model. Liquid quench simulations by Tomas et al.^61^ using a machine learning GAP potential at 3.0 g/cc and 3000 K for 200 ps appear to be consistent with the current DFT results. Tomas et al. also compare results of liquid carbon quench simulations for numerous force fields available in the literature^36,61^. However, a comparison with long-time large-scale DFT simulations, as shown here, is lacking.

**Figure 5:**
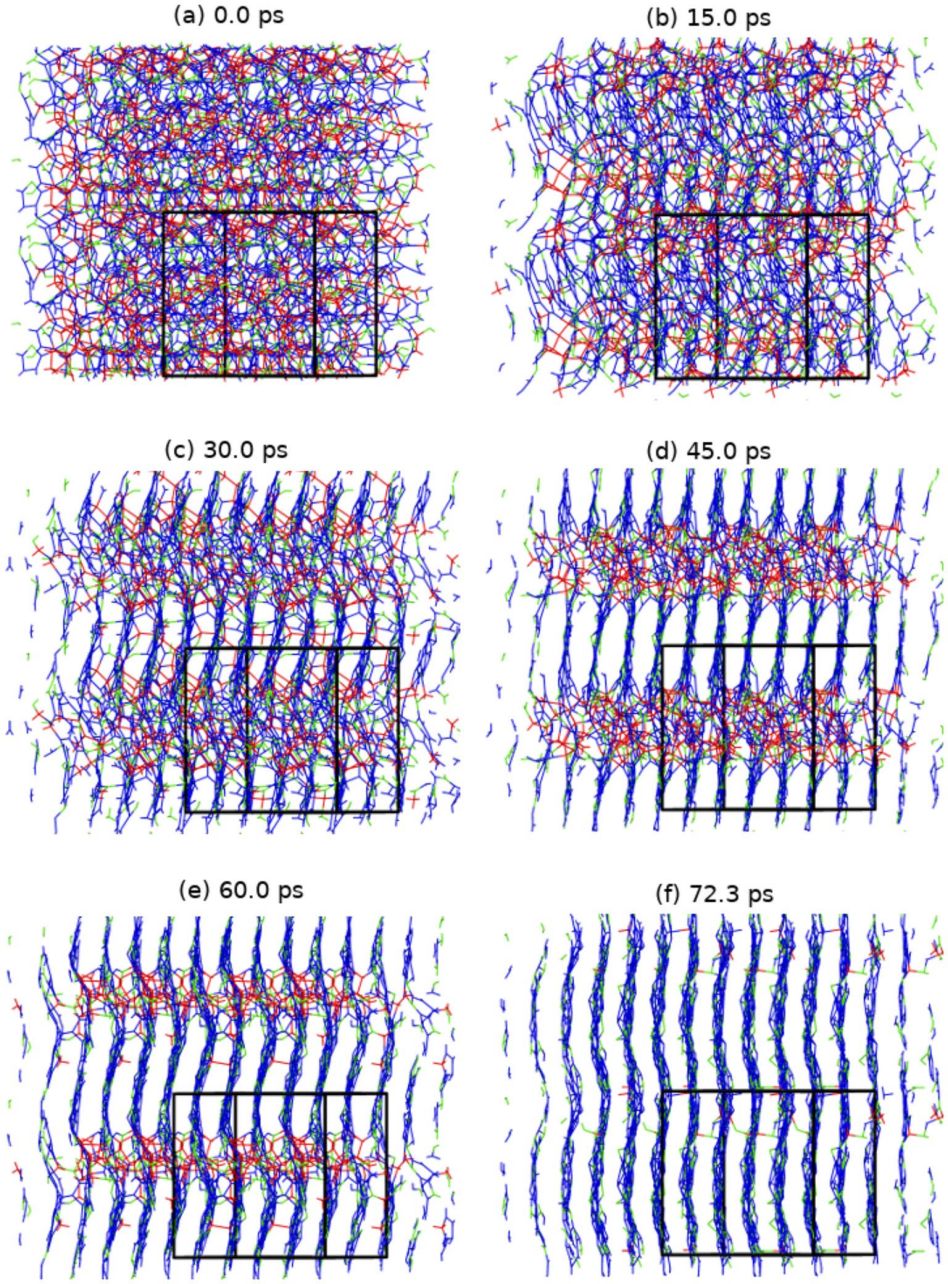
Structural evolution as a function of time of a-CN at 3000 K and 2.8 g/cc at 0.0, 15.0, 30.0, 45.0, 60.0, and 72.3 ps showing the transformation to a layered structure. Same coloring scheme as Fig 4 was used except for nitrogen atoms which are green. For visualization purposes, each image is a 2×2×2 supercell of the actual simulation cell (black box).

Snapshots of the final simulation configurations - see Fig. 3 and SI Figure S4, show the distinct structural properties of the system at the different densities studied. In particular, the presence of layered structures is visually striking for both the a-C and a-CN simulations up to 2.9 g/cc and 3.0 g/cc, respectively, although there are also obvious differences between the systems in this region. At densities of 2.3 and 2.7 g/cc the a-C simulations exhibit uniform layering characteristic of graphite, but at 2.9 g/cc extended non-layered high density regions are also present, while the 1.9 g/cc structure appears only marginally layered with large voids. The corresponding a-CN structures are reminiscent of the a-C ones, but the 2.4 g/cc structure displays non-negligible interlayer connectivity compared with its a-C counterpart and 2.0 g/cc shows no layering at all. At the same time, nitrogen appears to be well embedded in the carbon structures, with some free molecular nitrogen only observed at the lowest densities (2.0 and 2.4 g/cc), as evidenced by the nitrogen-nitrogen radial distribution functions (RDF) (SI Figure S5) with the characteristic N_2_ molecular bond peak at $\simeq$1.1Å. Interestingly, at 2.8 g/cc and 3.8 g/cc the nitrogen-nitrogen RDF exhibits a peak at $\simeq$1.34 Å, which is also present but weaker at other densities. It is worth noting that this distance is between that of a single and double nitrogen-nitrogen bond (1.45 Å in hydrazine and 1.25 Å in trans-diimine) and close to the DFT-calculated bond lengths of aromatic all-nitrogen rings (1.30 – 1.36 Å), predicted to be stable at high pressures (>15 GPa)^81–83^. We note that nitrogen-nitrogen bonds undermine the stability of N-doped graphene and other carbon-nitrogen structures^84,85^.

**Figure 6:**
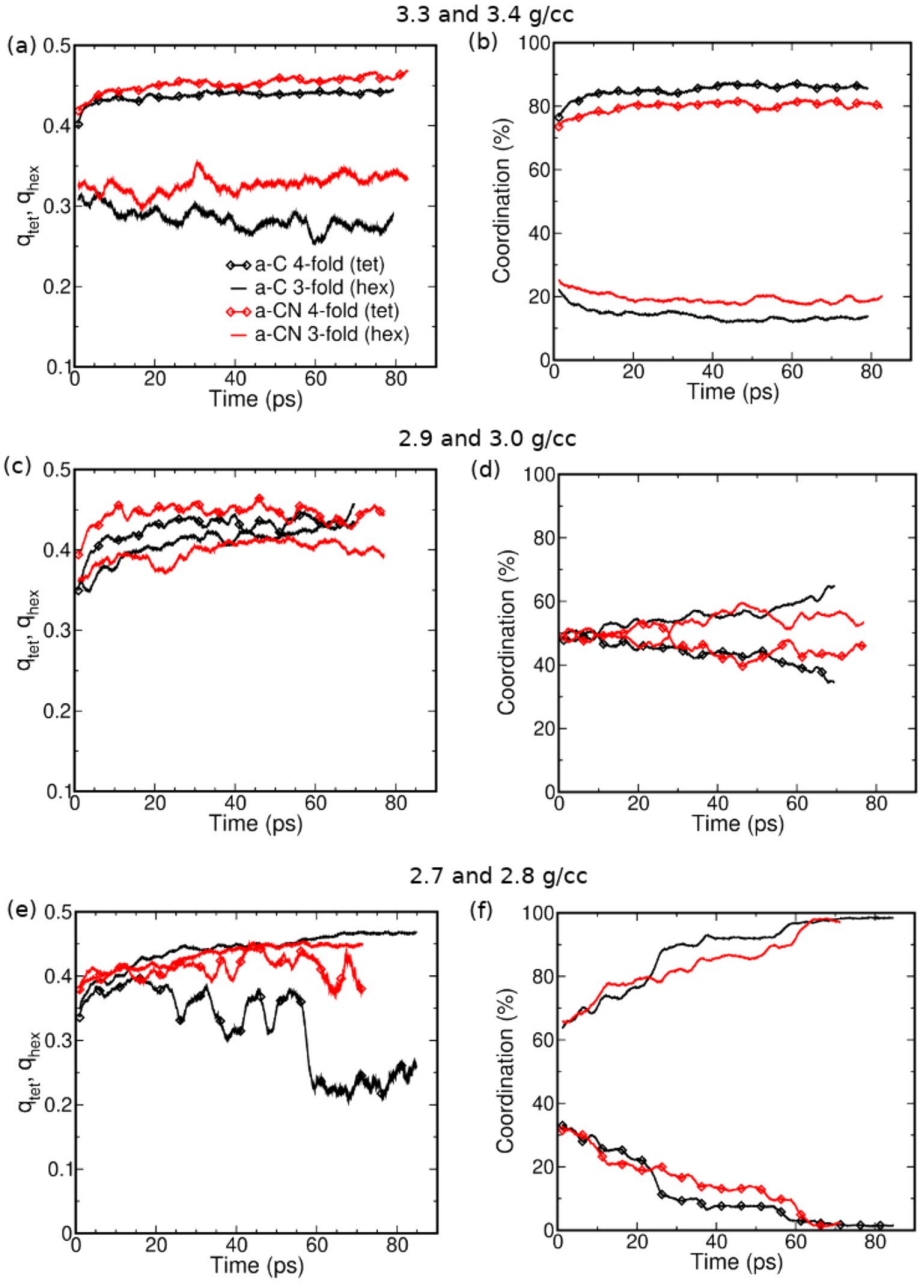
The tetrahedral (lines with diamond symbols) and hexagonal (lines with no symbols) order parameters (q$_{tet}$ and q$_{hex}$ respectively) and sp^3^ and sp^2^ fractions plotted as a function of time for a-C (black lines) and a-CN (red lines) at 3.3/3.4 (top), 2.9/3.0 (middle), and 2.7/2.8 g/cc (bottom) where the material is sp^3^ dominant, mixed, and sp^2^ dominant.

A major feature of the present simulations is that they exhibit clear time evolution of the system bonding and structural properties over 10’s of ps. This is evident for example in snapshots of the a-C (and a-CN) simulations at 2.7 g/cc (2.8 g/cc, respectively) - Figs. 4 and 5, as well as for those at 2.9 g/cc (3.0 g/cc, respectively) - SI Figures S6 and S7. However, the nature of the kinetics appears to be different at the low (and high) densities compared with the intermediate density region. To better understand the features of the unfolding kinetics we analyzed the fractions of 3-fold and 4-fold coordinated carbon atoms as a function of time, and calculated average local order parameters characterizing tetrahedral ($q_{tet}$) and planar hexagonal ($q_{hex}$) arrangements associated with these bonding states, respectively^86^. Order parameter values between 0.3 and 1 indicate structural order, with 1 corresponding to the ideal structures. The results are plotted in Fig. 6 for the a-C system at 2.7, 2.9, and 3.3 g/cc, and a-CN at 2.8, 3.0, and 3.4 g/cc.

**Figure 7:**
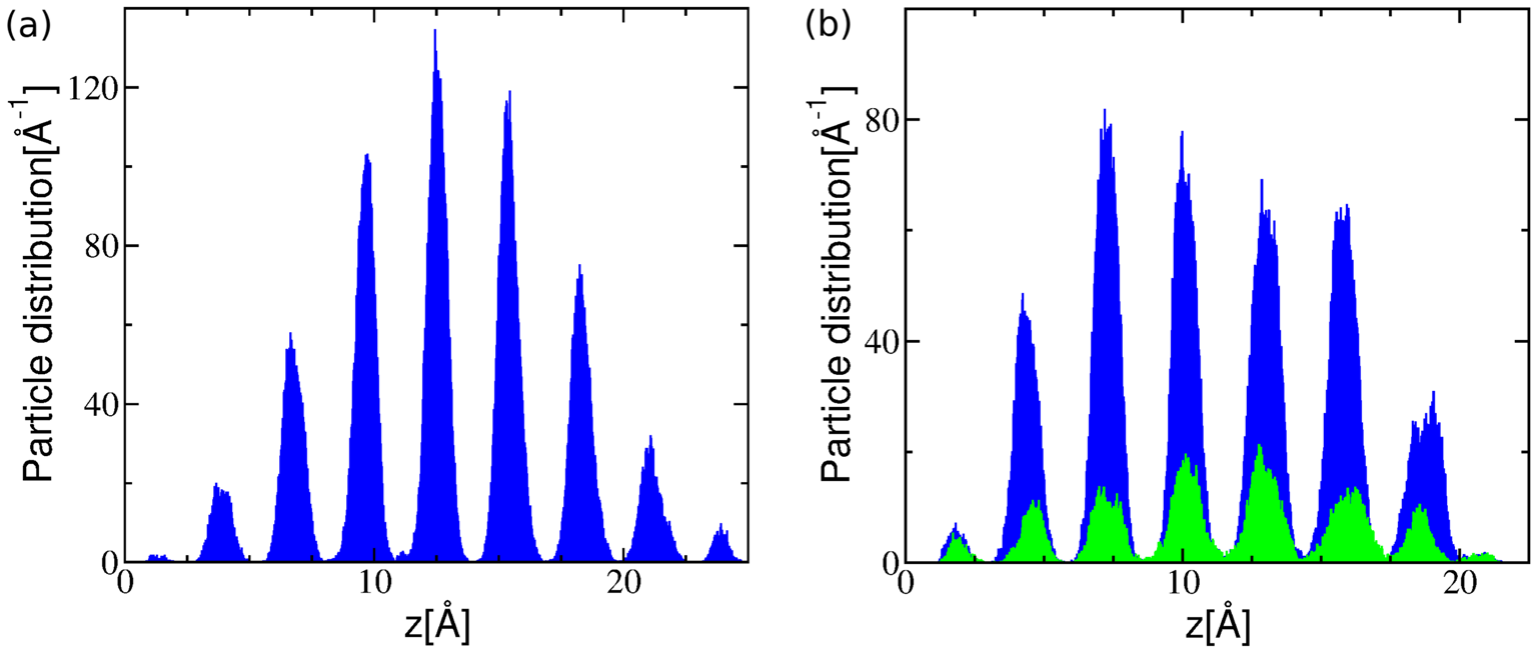
Particle distribution along a direction perpendicular to the layers of (**a**) a-C at 2.7 g/cc and of (**b**) a-CN at 2.8 g/cc (carbon atoms - blue, nitrogen atoms - green). Average interlayer distance is 2.88 Å.

**Figure 8:**
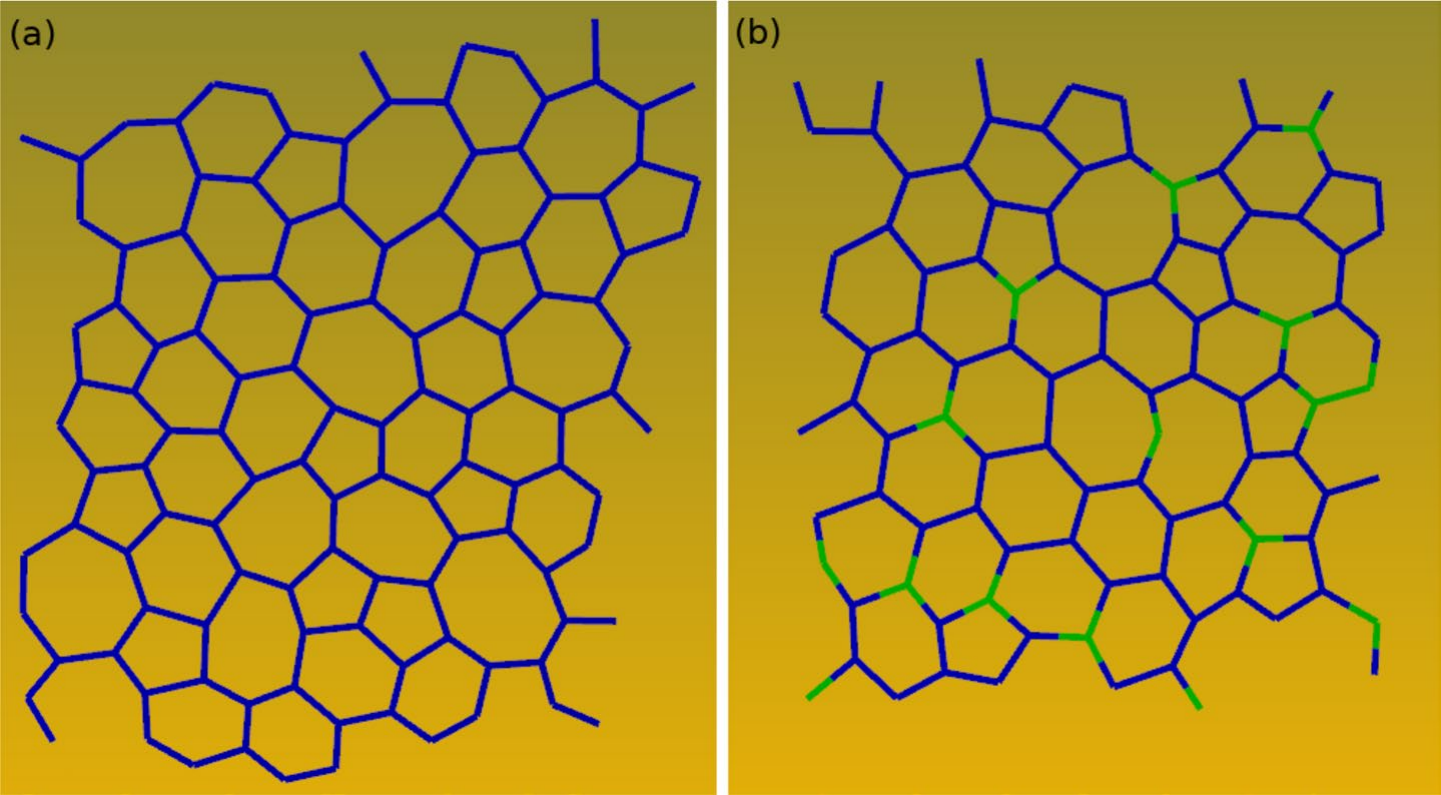
Bonding structure of a single layer for (**a**) a-C at 2.7 g/cc and for (**b**) a-CN at 2.8 g/cc.

At 2.7 g/cc the a-C fraction of 3-fold coordinated carbons increases rapidly following the temperature quench to encompass essentially the full system in approximately 60 ps, and the number of 4-fold coordinated atoms correspondingly decreases to almost zero. At the same time, the structural order parameter $q_{hex}$ describing planar sp^2^ arrangements steadily rises, while $q_{tet}$ signals only marginal then complete loss of tetrahedral order. This is confirmed by the system snapshots - see Fig. 4, which show well defined 4-fold coordinated domains shrinking as the background layered graphitic order becomes more pronounced. Analysis of the late times structure shows regular layering - see Fig. 7a and yields an interlayer distance for the resulting material of 2.88 Å; this is identical with the value for graphite at this density and 300 K^87^. Thus, at the current conditions intralayer bonding defects are the dominant system structural disorder feature, as already suggested by $q_{hex}$, albeit layer corrugation is also noticeable. A close look at a single layer - see Fig. 8a, reveals a disordered lattice consisting of distorted 5, 6, 7, and 8-member rings. Although the system size is too small for detailed comparisons, the structure appears similar with that of recently synthesized monolayer amorphous carbon (MAC)^45^. At the same time, the layer thickness and interlayer distance are approximately half that of the MAC and multilayer MAC, respectively; we attribute this to the high bulk density of the current material. It may be interesting to see if a single suspended layer experiences buckling and a corresponding increase of its height profile once it is exfoliated from the present system; this will require larger scale simulations. We note that in this density range the present results are in close agreement with the ab initio simulations of Thapa et al.^41^.

No nucleation effects are obvious in the simulations, with domains of identifiable phases emerging almost spontaneously (in less than 10 ps) following the quench. The subsequent ordering seems to consist of two distinct stages: in the first one the diminishing 4-fold regions are larger than the graphitic interlayer distance, while in the second one the remaining 4-fold coordinated carbons serve as independent tetrahedral interlayer bridges that gradually vanish. We speculate that at these thermodynamic conditions, which should be close to the edge of graphite - diamond coexistence, the first stage of the interphase boundary motion is driven by its curvature, as in classical ordering with a non-conserved order parameter^88^. While the system size is too small to fully resolve this issue, the early time evolution of the number of bonds between 3-fold and 4-fold atoms (N_34_), which we employ as a proxy for the interface area, seems to be consistent with this mechanism - see SI Figure S5. Motion by curvature generates a characteristic asymptotic growth law for the size of the domains, $R\propto t^{\frac{1}{2}}$^88,89^. The current simulations show that graphitic domains of order 1 nm are reached in 10’s of ps, which implies that in systems of $\simeq$10 nm the transformation should require a few nanoseconds. Although direct experimental measurements are not available, experiments characterizing the precipitation of $\simeq$10 nm liquid carbon droplets during a detonation event and their solidification into graphitic nano-onions^77^ suggest that the transformation occurs on time-scales that are at least an order of magnitude smaller that those of the associated adiabatic expansion, i.e. $\lesssim$10 ns. Thus, the present results are likely consistent with experimental observations and can be used to inform larger scale simulations of this process employing well-trained empirical force-fields^51,52^. They are also in qualitative in agreement with detonation experiments on hydrocarbons and oxygen mixtures, which produce graphene nanosheets^90,91^.

The addition of nitrogen (a-CN at 2.8 g/cc) changes subtly the carbon bonding kinetics - see Fig. 6, by slightly reducing the majority 3-fold fraction and increasing the 4-fold one. At the same time, nitrogen appears to support the tetrahedral structural order of the shrinking 4-fold coordinated phase, which persists for a long time as an interlayer reinforcement - see Fig. 5. The effect is even more pronounced at 2.4 g/cc, which displays strong interlayer connectivity compared with its 2.3 g/cc a-C counterpart. The interlayer spacing of the resulting material is approximately identical with that of a-C (at 2.7 g/cc), with most nitrogen atoms well distributed within the layers - see Fig. 7b, although the layer “thickness”, as measured by the width of the particle distributions, appears to be approximately 9% larger. The single layer structure is also similar - see Fig. 8b, but the amount of intralayer bonding defects is higher for a-CN (2.8 g/cc) than a-C (2.7 g/cc), as evidenced by the larger ratio of 5-membered to 6-membered rings in the final structures - see SI Figure S10. This contributes to a reduction in the first peak of the carbon-carbon RDF of a-CN compared to a-C - see SI Figure S11. The enhanced intralayer bonding defects of a-CN at 2.8 g/cc are consistent with the reduction of the $q_{hex}$ order parameter compared with a-C - see Fig. 6e. At the same time, the minority tetrahedral phase exhibits more ordering in a-CN, which also contributes to the structural differences between a-C and a-CN.

We note that layered a-CN structures have potential applications as electrocatalysts for oxygen reduction reactions because the N-doped carbons enhance electrocatalytic activity compared to platinum-loaded carbon electrodes^9,92–94^. The spin density and charge distribution near N-doped regions have been reported to be responsible for activated regions involved in catalytic reactions on N-doped graphene surfaces^95–97^.

**Figure 9:**
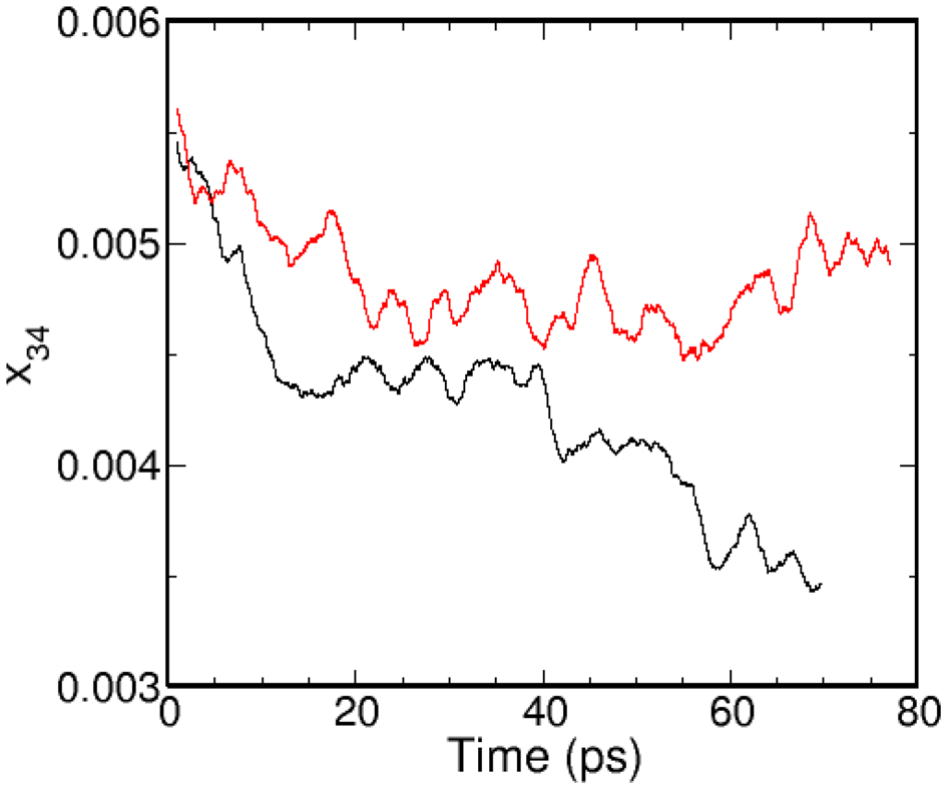
The change in the scaled number of bonds between 3-fold (sp^2^) and 4-fold (sp^3^) carbon atoms as a function of time at 2.9 g/cc for a-C (black lines) and 3.0 g/cc a-CN (red lines).

The a-C simulation at 3.3 g/cc shows that at this density the 4-fold coordinated tetrahedral order is favored over the 3-fold planar one - see Fig. 3. However, the number of 4-fold carbons seems to plateau quickly on the time scales of the simulations, while at late times $q_{tet}$ increases only very slowly, if at all. The same effect is observed at a higher density of 3.7 g/cc (not shown), where although the sp^3^ fraction is slightly larger, tetrahedral ordering as quantified by $q_{tet}$ appears to asymptote to a similar value. For both densities, small but non-negligible fractions of 3-fold carbons in locally disordered arrangements remain present for the duration of the simulations - see Fig. 6. When compared with the marked graphitic layering and the almost complete disappearance of sp^3^-bonded atoms observed at 2.7 g/cc, this behavior suggests that fast temperature quenches inhibit structural ordering more efficiently at high densities (and pressures) than at lower ones. This is likely a consequence of the much slower diffusive motion occurring at these conditions - see SI Figure S6. a-CN simulations at 3.4 g/cc show evolution of the coordination fractions similar with the a-C results, but again somewhat reduced majority (4-fold) and increased minority (3-fold) populations, along with more persistent order of the minority phase, as evidenced by $q_{hex}$ - see Fig. 6. The latter effect is comparable with the one observed at 2.8 g/cc, although the minority and majority coordinations are reversed here.

The intermediate density (2.9 g/cc) a-C simulations display distinguishing characteristics compared with the ones at lower and higher densities, as can be observed in SI Figure S6. Regions of sp^3^ and sp^2^ coordination, the latter showing significant layering, develop during the simulation, but their relative spatial extent seems to remain almost constant. This is confirmed by the coordination fractions, which change slowly over the course of the simulations - see Fig. 6. Moreover, in contrast with the low and high density cases, the order parameters $q_{tet}$ and $q_{hex}$ both increase with time, indicating continuing structural ordering of the sp^3^ and sp^2^ regions. To better understand the nature of this process we calculated the (scaled) number of bonds between sp^3^ and sp^2^ carbons, $x_{34}=N_{34}/(N_3 N_4)$ (here $N_4$ and $N_3$ are the number of sp^3^ and sp^2^ atoms, respectively); this is plotted in Fig. 9. The result shows that $x_{34}$ decreases in time, suggesting that the sp^3^ and sp^2^ domains are separating and growing in time, although larger scale simulations are necessary to fully elucidate the kinetics. The corresponding a-CN system at 3.0 g/cc is visually somewhat more disordered than the a-C system - see SI Figure S7, although both the coordination fractions and order parameters display similar behavior. This is supported by the evolution of $x_{34}$, which remains largely constant rather than decrease in time - see Fig. 6. Thus, nitrogen appears to inhibit the growth of diamond-like and graphite-like domains, thereby promoting amorphous character. This observation is supported by the evolution of the sp^2^ coordination - Fig. 6d, which increases for a-C but not a-CN, and is also consistent with the previously noted (see Fig. 1) apparent reduction of the volume change between the low and high pressure phases. To further quantify the role of nitrogen in determining the kinetic evolution of the a-CN systems we calculated the number of nitrogen - carbon bonds for all densities studied, see SI Figure S12. These are essentially time-independent, implying that carbon-bonded nitrogen remains trapped in the carbon structures over time-scales of order 100’s of picoseconds and likely longer. The effect may be observable during fast temperature quenches, such as those associated with detonation events, $\gtrsim 10^9$ K/s^77^.

## Methods

Ab initio MD simulations were performed with the Vienna Ab initio Simulation Package (VASP)^98–100^. The Perdew-Burke-Ernzerhof (PBE)^101^ exchange-correlation functional under the generalized gradient approximation (GGA) was used along with projector-augmented wave (PAW) pseudo-potentials^102,103^. To account for the van der Waals forces, the Grimme semi-empirical dispersion correction^104^ was also employed. A plane wave basis set with an energy cutoff of 450 eV was used along with a k-point spacing of 0.07 Å$^{-1}$. This means that the 512 atom simulations were at the $\Gamma$ point in reciprocal space.

Constant temperature and volume (NVT) simulations were performed using the Nosé-Hoover thermostat with a characteristic mass corresponding to a 20 fs relaxation time^105,106^. The timestep was set to 0.5 fs and the self consistent field (SCF) convergence threshold was set to $10^{-7}$ eV. A Fermi smearing of electronic states was used with a width equal to $k_{B} T$, where $k_{B}$ is the Boltzmann constant. These settings were chosen so the simulations would be conservative at the highest temperature that we considered, 9000 K.

The starting structure for the simulations was liquid carbon (LC) taken from a previous study that used 64 atoms^107,108^. LC at densities between 1.9 and 3.7 g/cc was thermalized at 9000 K for 10 ps. The temperature was then instantly reduced to 6000 K and  the system was thermalized for another 10 ps. We note that at these elevated temperatures LC reaches equilibrium very rapidly. The pressure-volume curves for LC and LCN at 6000 K and 9000 K were smooth for the full pressure range, see SI Figure S1. The 64 atoms structure was then enlarged to 512 atoms to decrease finite size effects and the temperature was instantly reduced to 3000 K. Simulations at this temperature were performed for > 65 ps for each density except 1.9 g/cc, which was ran for 46 ps. Simulations with nitrogen content (thereafter denoted by LCN) were performed by randomly replacing 13 carbon atoms (or 20.3%) of the original 64 atoms LC structure with nitrogen atoms and repeating the same procedure described above starting with thermalization at 9000 K. The choice for the nitrogen concentration was loosely based on previously reported results for C-N systems as well as the properties of carbon materials recovered from detonation events^50^.

## Conclusions

In summary, we performed ab initio MD simulations of pure (a-C) and nitrogen-containing (20.3% N) (a-CN) amorphous carbon formation at high pressures and temperatures following a rapid quench from a liquid state to a solid state region. The results show that the system properties are determined by the competition between $sp^2$ and $sp^3$ bonding, which unfolds over 10’s of picoseconds time scales and is therefore important even for very high quenching rates. We find that $sp^2$ and $sp^3$ hybridizations dominate at low and high densities, respectively, and are associated with distinct structural characteristics typical of layered (graphite-like) and tetrahedral (diamond-like) order. The layered graphite-like material is similar with the one studied by Thapa et al.^41^, which the authors call amorphous graphite (a-G), while the tetrahedral one can be identified as diamond-like amorphous carbon (DLC). a-G exhibits the regular layering of graphite, but with significant intralayer disorder characterized by the presence of deformed 5, 6, 7, and 8-member rings, resembling monolayer amorphous carbon (MAC)^45^. At intermediate densities the $sp^2$ and $sp^3$-bonded carbon atoms coexist in recognizable graphitic and diamond-like domains that appear to grow in time for the pure carbon system, while the growth is kinetically inhibited when nitrogen is present. In this region the system, which we dub structurally-mixed amorphous carbon (SMAC), exhibits high compressibility inherited from the underlying diamond - graphite phase coexistence, which will affect its response to applied pressures even on sub-nanosecond time scales. The simulations indicate that the transformation from the liquid state to a graphitic solid state can proceed to completion for nanometer size systems in a fraction of a nanosecond, possibly via a mechanism controlled by interphase curvature. This is consistent with available experimental results that detect the solidification of $\simeq$10nm liquid carbon nano-droplets produced by detonation events into graphitic nano-onions over much shorter time scales, $\lesssim$10 ns, than those of the associated adiabatic expansion. The addition of nitrogen to the system has the surprising effect of supporting rather than disrupting the two dominant carbon structural motifs, tetrahedral and layered, even for the fairly high nitrogen concentration studied here, 20.3%. This is particularly striking for the minority phases, leading for example at lower densities to the formation of graphitic structures with persistent tetrahedral interlayer reinforcement. The resulting material has similar features with a-G, with nitrogen well intercalated in the disordered layers. No elimination of carbon-bonded nitrogen appears to occur at high densities during simulations running close to 100 ps, suggesting that at high pressures nitrogen is likely to remain embedded in the carbon structure during fast temperature quenches. The present results provide direct information on the structure and dynamics of amorphous carbon (a-C and a-CN), can serve as reference points for the generation of effective force fields capable of in silico carbon materials engineering and evaluation, and may be useful for the development of effective equations of state and kinetic models for high temperature and pressure phenomena, such as ion beam deposition, laser ablation, high pressure synthesis, shock-induced chemistry, and detonation.

### Supplementary Information

Supplementary Information 1.

## Data Availability

The datasets generated and/or analysed during the current study will be made available in the repository at https://nomad-lab.eu/.
