# Supplementary material for: Ab initio structural dynamics of pure and nitrogen-containing amorphous carbon
Source: Sci Rep. 2023 Nov 11;13:19657. doi: 10.1038/s41598-023-46642-7 (PMC10640601; doi:10.1038/s41598-023-46642-7)
Supplement: Supplementary file 1 — Supplementary Information 1. [file 41598_2023_46642_MOESM1_ESM.pdf]

Supporting Information: Ab initio Structural Dynamics of Pure and Nitrogen-containing Amorphous Carbon

Brad A. Steele, Sorin Bastea,* and I-Feng W. Kuo

Lawrence Livermore National Laboratory, Physical and Life Sciences Directorate, P.O. Box 808, Livermore, California 94550, USA

E-mail: bastea2@llnl.gov

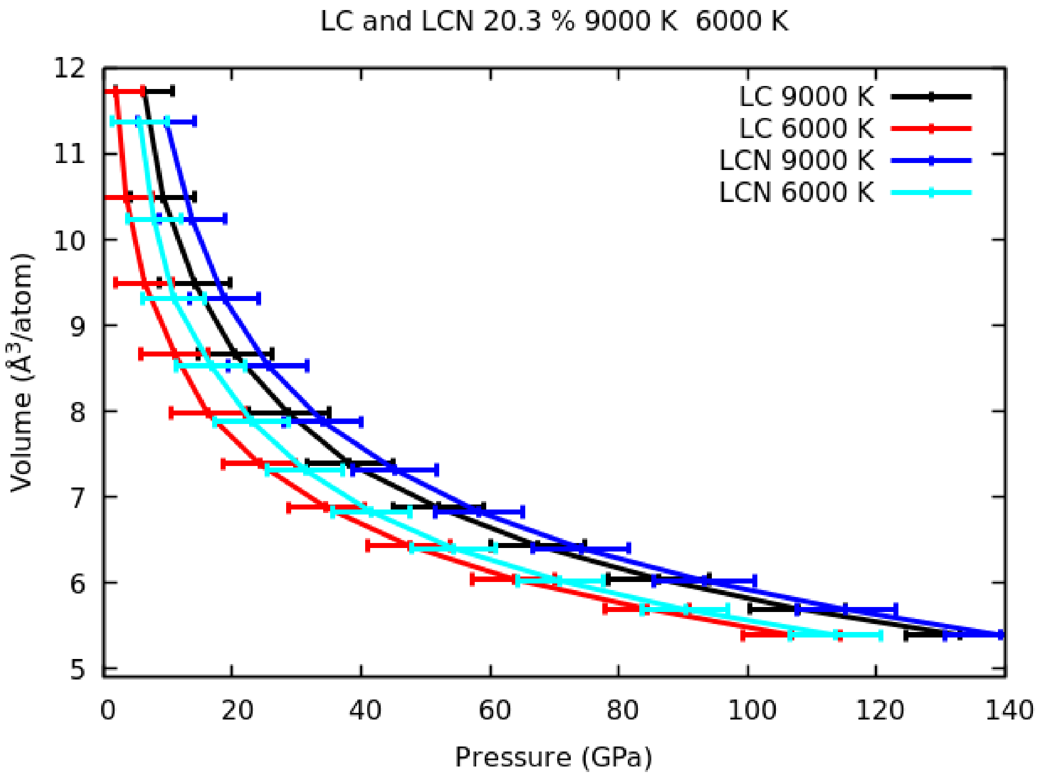

Figure S1: Pressure-volume relationship for liquid carbon (LC) and liquid carbon/nitrogen (LCN) mixture (20.3 % N) at 9000 K and 6000 K. At each temperature and density the system was thermalized for 10 ps, and the pressures were averaged over the final 5 ps of the simulation.

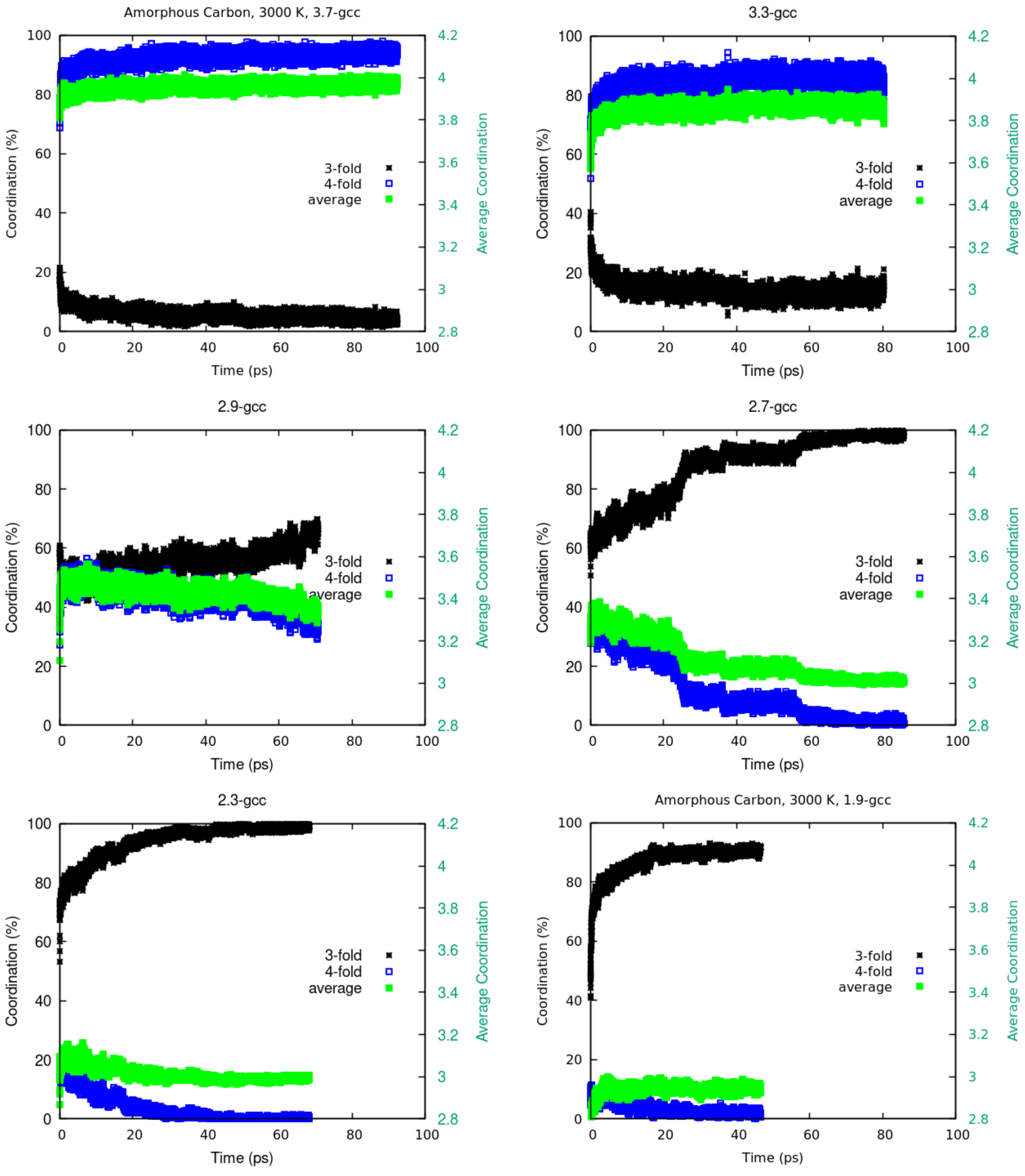

Figure S2: Coordination vs. time for amorphous carbon (a-C) at 3000 K.

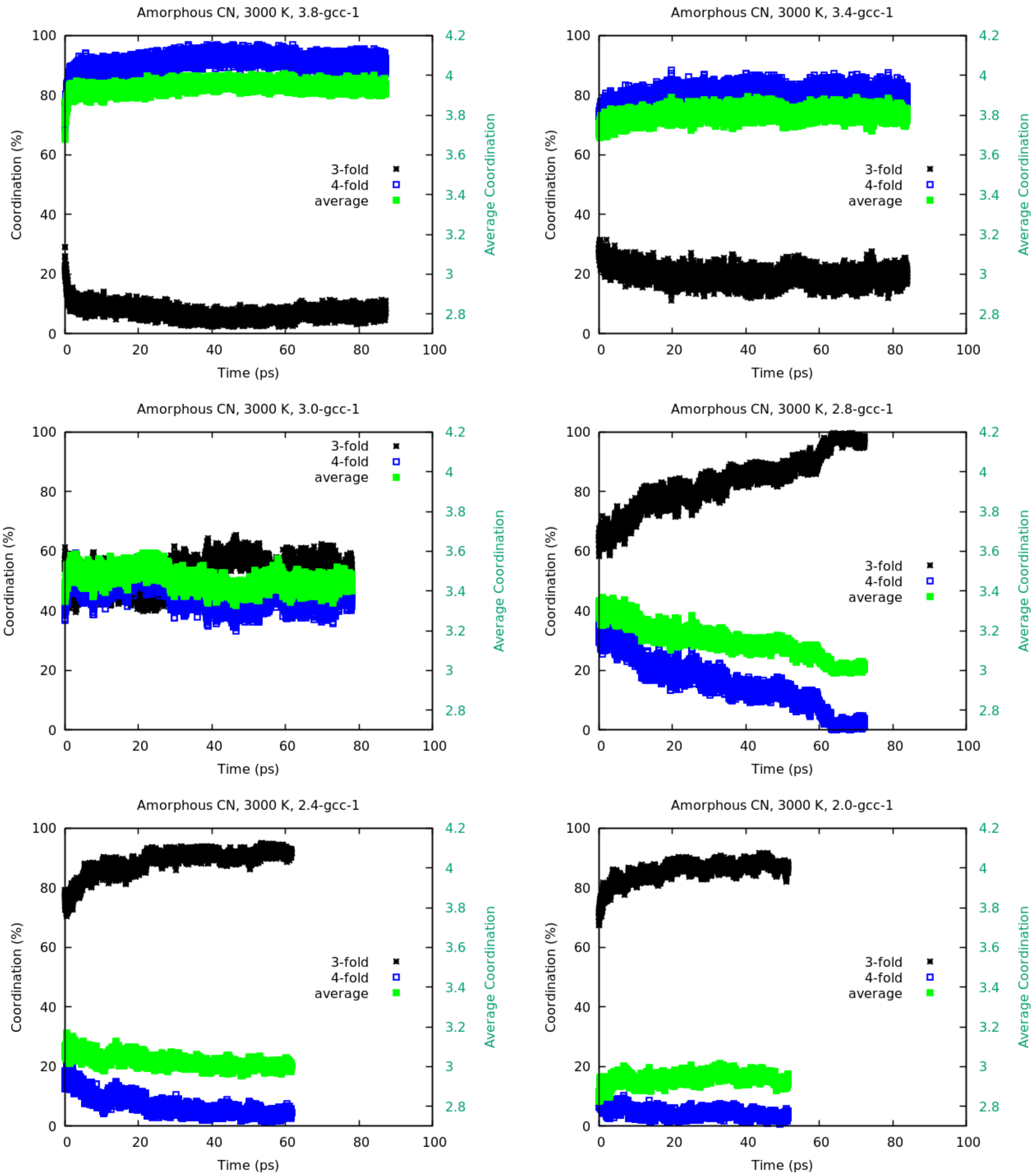

Figure S3: Coordination vs. time for nitrogen-containing amorphous carbon (a-CN) at 3000 K.

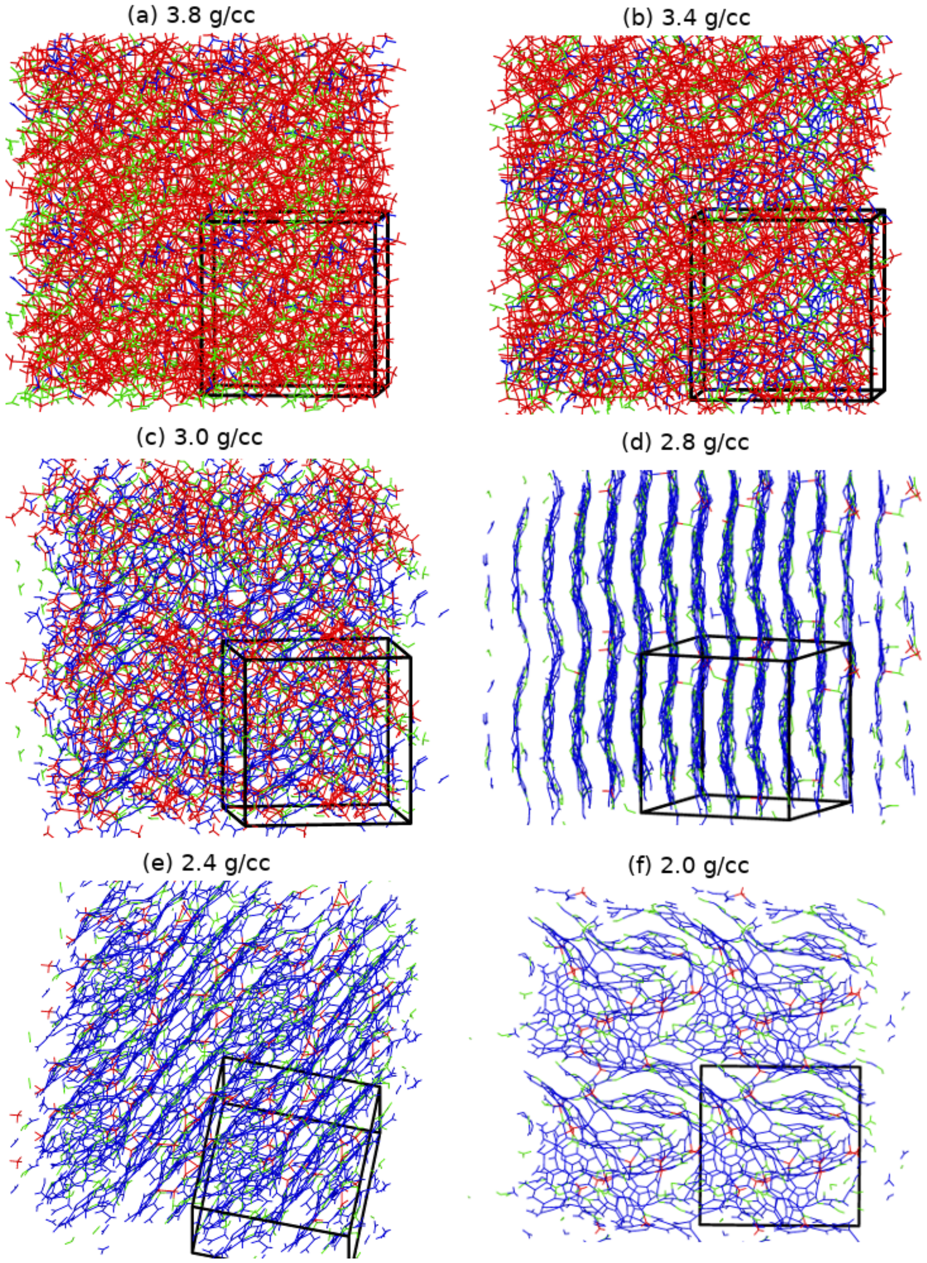

Figure S4: Snapshots of the final structure of a-CN at 3000 K at each density studied. Carbon atoms are color-coded based on the atomic coordination; 3-fold atoms are blue, 4-fold atoms are red, and nitrogen atoms are green. For visualization purposes, each image is a 2x2x2 supercell of the actual simulation cell (black box).

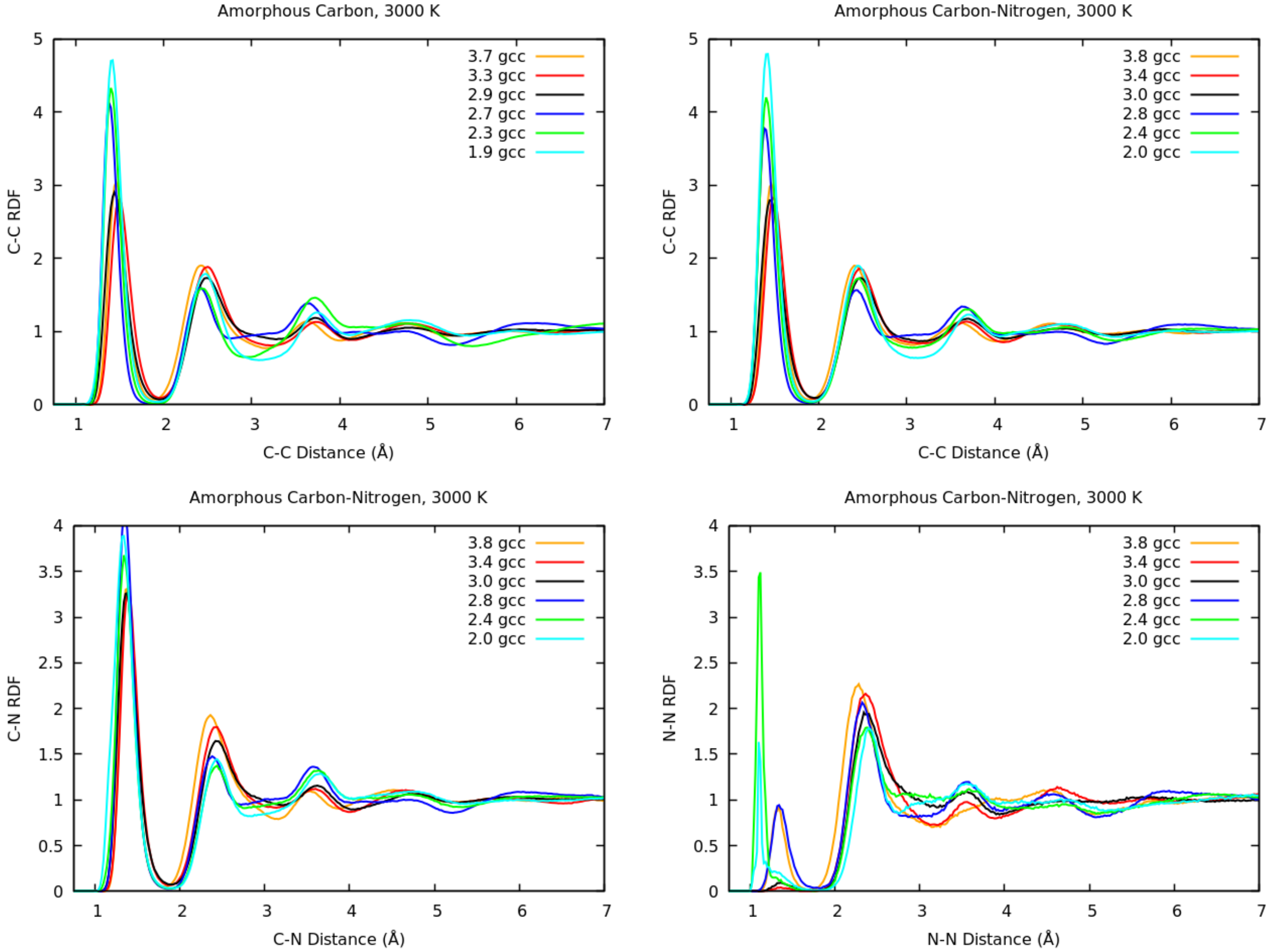

Figure S5: Pairwise radial distribution functions (RDF) for all densities studied of a-C and a-CN at 3000 K.

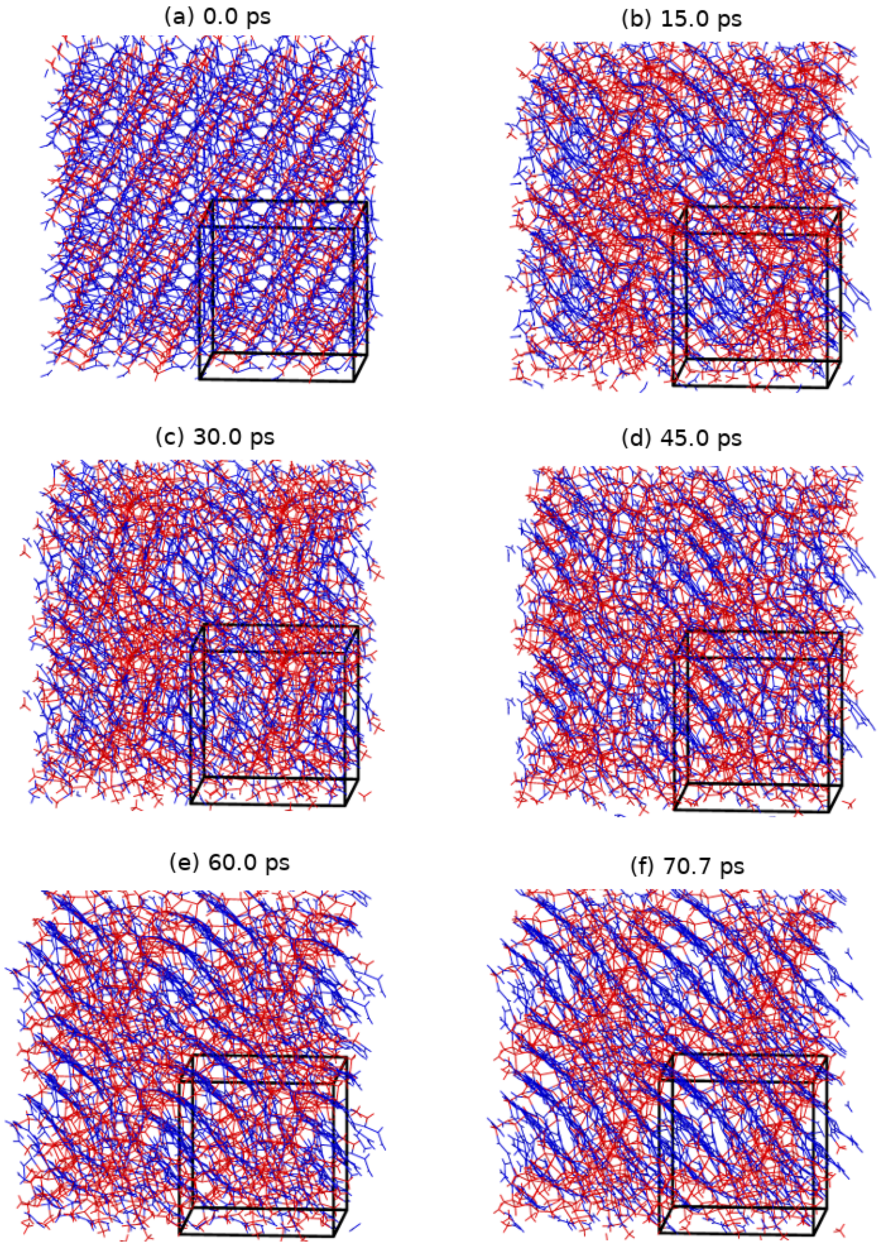

Figure S6: Structural evolution as a function of time of a-C at 3000 K at 2.9 g/cc at 0.0, 15.0, 30.0, 45.0, 60.0, and 70.7 ps. Atoms were color-coded based on the atomic coordination. For visualization purposes, each image is a 2x2x2 supercell of the actual simulation cell (black box).

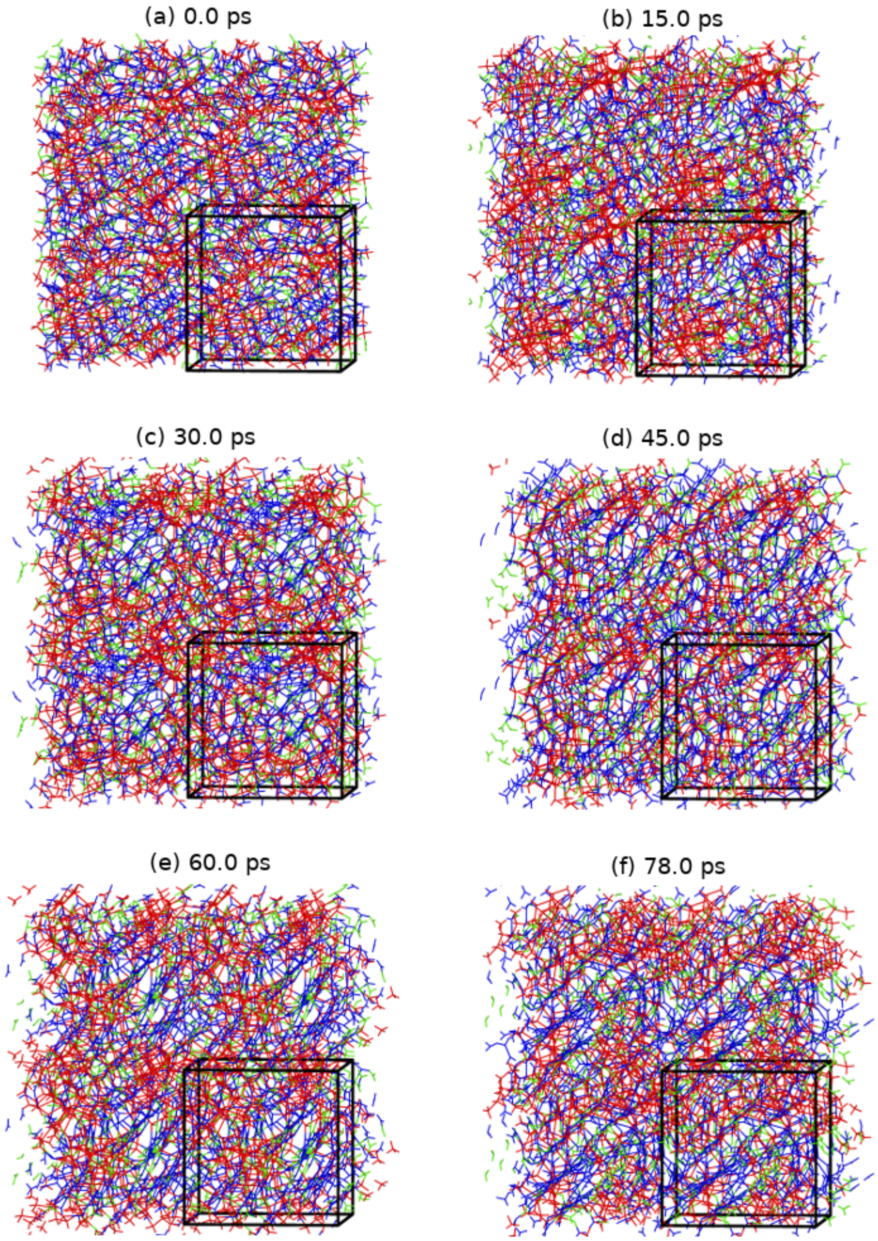

Figure S7: Structural evolution as a function of time of a-CN at 3000 K at 3.0 g/cc at 0.0, 15.0, 30.0, 45.0, 60.0, 78.0 ps. Atoms are color-coded based on the atomic coordination, except for nitrogen atoms which are green. For visualization purposes, each image is a 2x2x2 supercell of the actual simulation cell (black box).

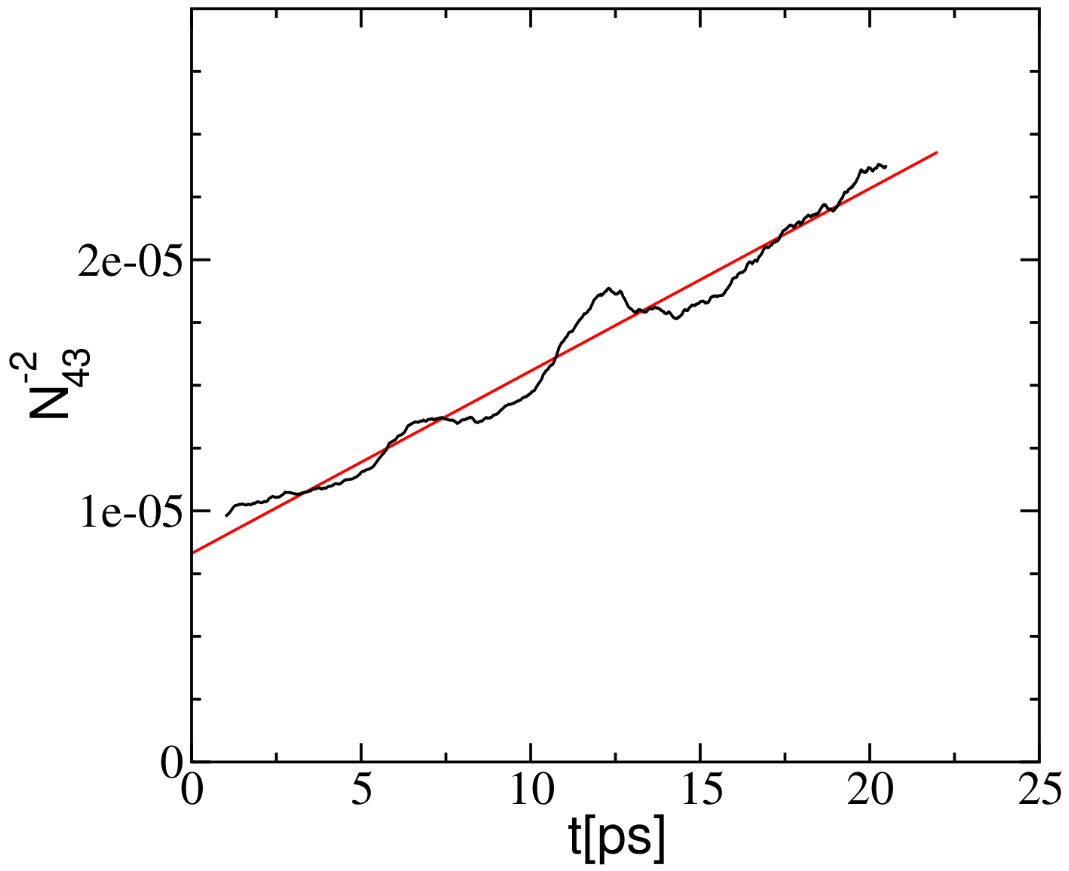

Figure S8: Time evolution of the number of bonds between 3-fold and 4-fold atoms (N_{43}), which is employed as a proxy for the interface area S between 3-fold and 4-fold regions. For domain growth driven by curvature S^{-2} increases linearly in time.

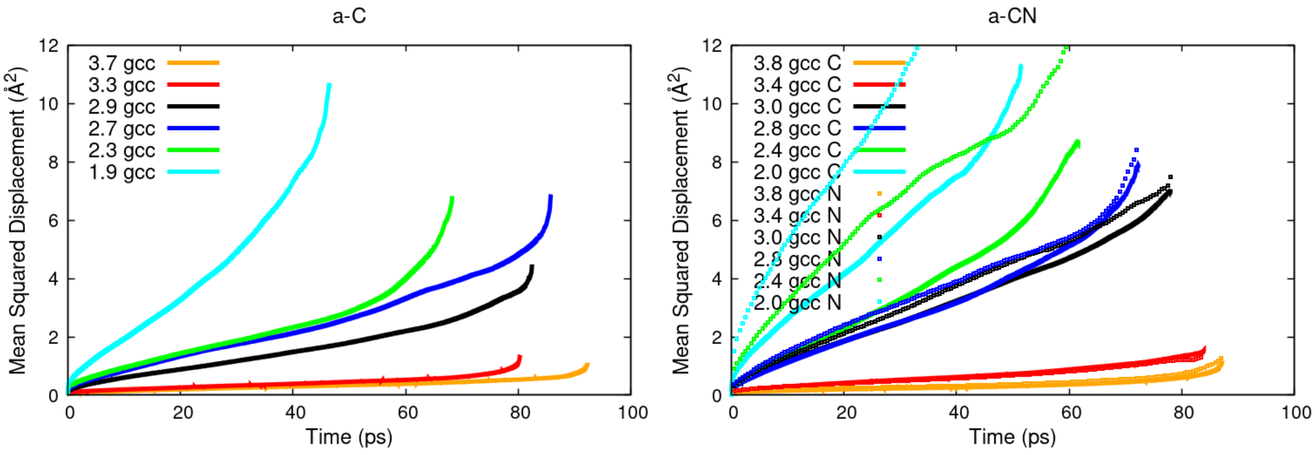

Figure S9: Mean square displacements (MSD) as a function of time for all densities studied of a-C and a-CN at 3000 K. For a-CN, the MSD was calculated individually for carbon atoms (lines) and nitrogen atoms (symbols) - the nitrogen atoms generally have a larger MSD. The tail end of the MSD is not reliable because there are not enough points to perform the average.

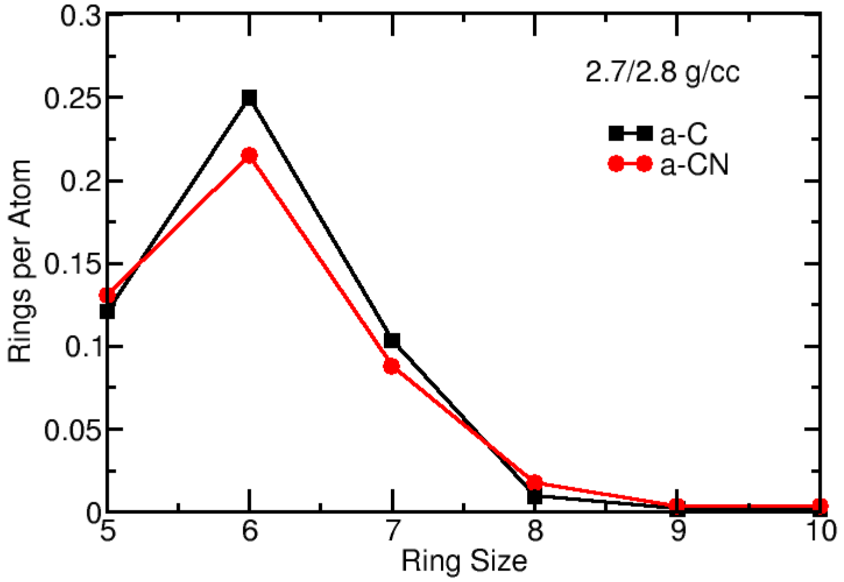

Figure S10: The number of rings per atom for the final snapshot of the a-C (2.7 g/cc) and a-CN (2.8 g/cc) simulations. The ratio of 5 to 6 - membered rings is 0.48 for a-C and 0.61 for a-CN.

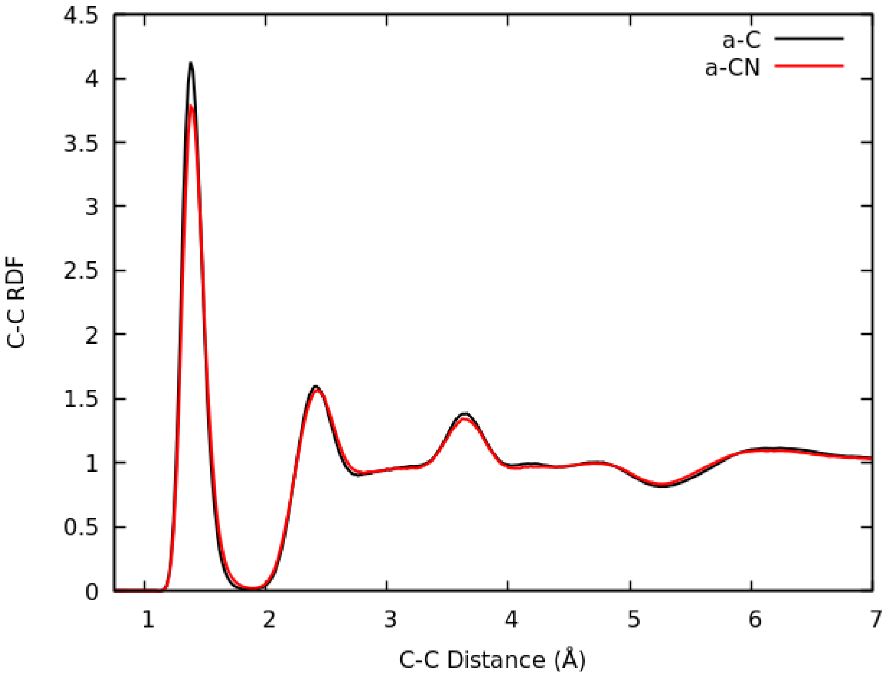

Figure S11: Comparison of the pairwise radial distribution functions (RDF) for the final 20 ps of the a-C and a-CN systems at 2.7 and 2.8 g/cc, respectively.

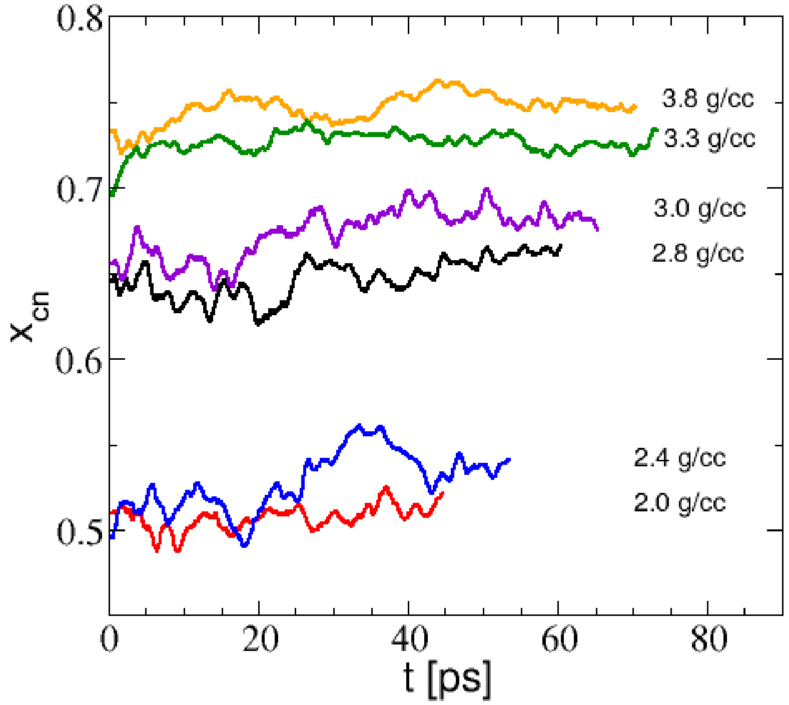

Figure S12: Number of carbon-nitrogen bonds vs. time at different densities.
